# Dimensions of Job Demands Among New-Generation Employees Based on Online Reviews by Employees

**DOI:** 10.3390/bs14110990

**Published:** 2024-10-24

**Authors:** Yanrong Hu, Lixin Zhou, Ping Huang, Qingyang Liu, Hongjiu Liu

**Affiliations:** 1 College of Mathematics and Computer Science, Zhejiang A & F University, Hangzhou 311300, China; yanrong_hu@zafu.edu.cn (Y.H.); huangping@zafu.edu.cn (P.H.); 2 Comprehensive Security Center of Hangzhou Xiaoshan District Development and Reform Bureau, Hangzhou 311300, China; lixinzhou2-c@my.cityu.edu.hk; 3 Institute of Informatics, Georg-August-Universität Göttingen, 37073 Göttingen, Germany

**Keywords:** online reviews, new-generation employees, job demand, dimensions, LDA, DEMATEL, ISM, MICMAC

## Abstract

Based on employees’ online reviews, this article analyzes the dimensions of job demand for new-generation employees, using a combination model of Latent Dirichlet Allocation, decision-making trials and evaluation laboratory, interpretative structural modeling, and cross-impact matrix multiplication (LDA—DEMATEL—ISM—MICMAC). The results show that job demand is composed of 10 dimensions, and there is significant interdependence between the dimensions. Changing one dimension will quickly affect the other dimensions. The dimension with the greatest influence degree is leadership and team atmosphere (*s*_1_), while the dimension with the highest affected degree and centrality degree is welfare and promotion (*s*_3_). Leadership and team atmosphere (*s*_1_), company culture and industry (*s*_4_), overall environment and platform (*s*_5_), and platform and technology (*s*_7_) were identified as the key factors. They play a causal role in job demand and have a significant impact on other dimensions. Dimensions such as working relationship and intensity (*s*_2_), welfare and promotion (*s*_3_), opportunity and resources (*s*_6_), business and industry development (*s*_8_), corporate prospects and personal development (*s*_9_), and work stress and position (*s*_10_) are affected by other dimensions and require special attention. The underlying need is present for the dimension of leadership and team atmosphere (*s*_1_), which has a direct or indirect impact on other dimensions in different ways.

## 1. Introduction

With the advanced development of digitalization and informatization, new-generation employees, especially millennials and Gen Z, are becoming the backbone of the workplace. They are driving rapid growth and the innovation of enterprises while redefining the value and meaning of work. This generation has grown up in a highly digital environment, leading to significant differences in their career perspectives and work expectations compared to previous generations. They pay more attention to work–life balance and value the realization of self-worth and social impact in their careers. They seek opportunities for innovation and growth at work, as well as an open and inclusive company culture, along with a positive work environment and interpersonal relationships. While traditional compensation and welfare remain important, these are no longer the sole driving forces behind their career choices.

For most enterprises, understanding and meeting the needs of new-generation employees are keys to attracting and retaining top talent, and these are also one of the core strategies to enhance the competitiveness of an enterprise. In a highly competitive and rapidly changing business environment, employee satisfaction and loyalty directly impact the company’s innovation capabilities and market performance. Therefore, it is of great theoretical and practical significance to systematically study the needs of new-generation employees at work.

Research on employee job demands has been extensively conducted, but most research has focused on traditional industries and classic motivational theories. Twenge and Campbell (2008) and Lyons and Kuron (2014) indicate significant differences in psychological traits and job demand among different generations of employees [1,2]. Particularly, new-generation employees are more concerned with work–life balance, self-development, and the meaning of work. Deloitte (2016) further analyzes the needs and expectations of millennial employees through surveys, suggesting how companies can attract and retain these new-generation employees. Although these studies provide a foundation for understanding employee needs, traditional methods and theories seem inadequate in the face of the unique characteristics of various industries and the special needs of new-generation employees. For this purpose, Morgeson and Humphrey (2006) developed the Work Design Questionnaire (WDQ), offering a more comprehensive tool for assessing job characteristics and employee needs [3]. Grant and Parker (2009) proposed a new theoretical perspective on work design, emphasizing relational and proactive aspects, which are crucial for understanding the needs of new-generation employees [4]. Kuvaas et al. (2017) studied the different impacts of intrinsic and extrinsic motivation on employee work outcomes, further deepening the understanding of employee needs and motivation [5]. Based on the above analysis, traditional needs analysis typically relies on surveys and interviews, which may not fully capture the true feelings and subtle changes in employee needs.

In this context, online reviews, as a new source of information, provide richer and more authentic data. These reviews reflect employees’ experiences and feedback in their actual work, with a high degree of authenticity and emotional nuance. By analyzing these online reviews, we can gain deeper insights into employees’ genuine views and needs regarding the work environment, corporate culture, management practices, and more, thereby more accurately understanding their expectations and satisfaction levels.

This paper employs the integrated model of LDA-DEMATEL-ISM-MICMAC to analyze the dimensions of the job demand for new-generation employees. The main objective of our research is to comprehensively and profoundly understand the diverse demands of new-generation employees. Specifically, our study aims to identify various dimensions of job demand for new-generation employees by analyzing online reviews, reveal the hierarchical structure and relationships among these demand dimensions, and provide organizations with personalized human resource strategies that carefully cater to their employees’ specific needs in order to better meet the unique demands of new-generation employees. In terms of research methodology, we use the LDA model for topic modeling, the DEMATEL model for identifying causal relationships among job demand dimensions, the ISM model for constructing a hierarchical structure of job demand dimensions, and the MICMAC model for analyzing the driving and dependence forces of each demand dimension. Our approach introduces a novel analytical framework that allows for a more dynamic and interactive understanding of employee demands. Additionally, we utilize online reviews from employees as a data source, providing a more candid and real-time reflection of employee emotions and needs, thus complementing the limitations of traditional survey-based methods. Through our research, we aim to refine and expand existing motivation theories, thereby contributing to the development of more effective theories and practices in human resource management.

## 2. Research Methods

**Figure 1:**
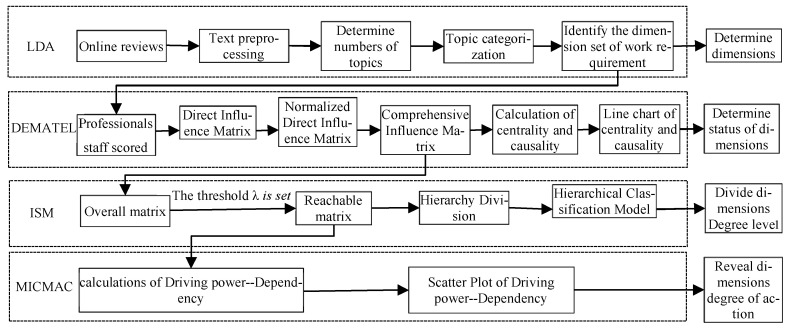
Research path diagram.

This paper employs the integrated model of LDA-DEMATEL-ISM-MICMAC to analyze the dimensions of the job demand for new-generation employees, with the specific research framework illustrated in Figure 1.

### 2.1. LDA Analysis

LDA is a document topic generation model, which is one of the classic topic mining models proposed by David Blei et al. This model is an unsupervised machine learning algorithm based on the Dirichlet distribution, which can be used to identify hidden thematic information in large-scale document sets or corpora [6]. LDA consists of three layers—words, topics, and documents—and is also referred to as a three-layer Bayesian probabilistic model [7]. It is a mixed probability model, where each document is represented as a probability distribution composed of multiple topics, and each topic is represented as a probability distribution composed of many words. The LDA model is often used in text classification, which can give the topic of each document in the document set in the form of probability distribution, allowing for topic extraction, topic clustering, or text classification based on the topic distributions.

**Figure 2:**
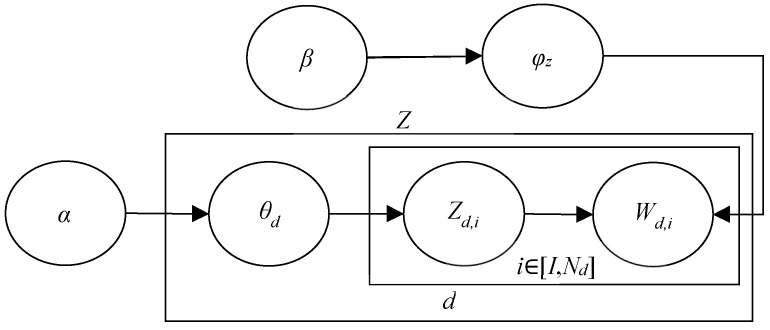
LDA topic model diagram.

This article constructs an LDA model to perform topic mining on review texts of job demand. As shown in Figure 2, *D* represents the content of the online reviews after data cleaning, *Z* represents the topic in the reviews, and both *α* and *β* represent the prior probability, which represents the strength of the potential topic and the probability distribution of the potential topic heading, respectively. *θ_d_* represents the matrix distribution of the relationship between the document and the subject, and *φ_z_* represents the matrix distribution of the relationship between the subject and the word [8]. *W_d,i_* denotes word vectors; the rectangular box on the periphery represents the repeated sampling process. In the LDA topic model, all reviews content is first aggregated into a document set to examine whether the review text *D* conforms to the multinomial distribution *θ* and to extract the corresponding topics *Z*. Next, the extracted topics *Z* are examined to satisfy the multinomial distribution *φ*, from which feature words *W* are further extracted. Finally, the above extraction process is repeated until every word in the review documents is processed [9].

### 2.2. Decision Making Trial and Evaluation Laboratory

The Decision-Making Trial and Evaluation Laboratory method (DEMATEL), proposed by Gabus and Fontela in 1971 [10], is a system decision analysis method that uses graph theory and matrix tools to interpret complex problems. By analyzing the logical relationship and direct influence relationship between the elements in the system, the method can judge the existence and strength of the relationship between the elements [11,12]. According to the mining and identification results of the LDA topic model, this paper uses the DEMATEL method to construct a network of the dimensions of job demand and calculates the causality degree and centrality of each dimension so as to quantitatively analyze the interaction and influence degree between these dimensions in the system. The specific calculation process is as follows:

(1) Determine the direct impact matrix *A*.

We used specific values (4, 3, 2, 1, 0) to quantify the strength of influence between the dimensions, where 4 was very strong effect, 3 was strong effect, 2 was moderate effect, 1 was weak effect, and 0 was no effect. We invited industry practitioners to assess the strength of the relationships between these dimensions, and the average value was taken to determine the direct impact matrix *A* = (*o_ij_*)*_n_*_×*m*_, where *n* is the matrix dimension; *o_ij_* is the influencing strength of dimension *F_i_* on the dimension in column *j*, and when *i* = *j*, *o_ij_* = 0.

(2) Calculate the comprehensive impact matrix *T*.

We normalized the direct impact matrix *A* to obtain the normalized matrix *N* [13], indicated as follows:

(1) $$N=\frac{O_{ij}}{max(\sum_{j=1}^{n}O_{ij})}$$

where *o_ij_* represents an element in the direct impact matrix.

The comprehensive impact matrix *T* (*T* = [*t_ij_*]*_n_*_×*n*_) is the sum of the direct and indirect effects between these dimensions.

(2) $$T=N^{1}+N^{2}+\ldots +N^{n}=\sum_{i=1}^{n}N^{i}$$

Since *o_ij_* ∈ [0, 1], when *n* → ∞, *o^n^*^−1^ → ∞, then there is

(3) $$T={N(I-N)}^{-1}$$

Here, *I* represents the identity matrix.

(3) Calculate the influence degree and affected degree of each dimension.

The expressions for the influence degree *D_i_* and the affected degree *C_j_* of each dimension are as follows:

(4) $$D_{i}=\sum_{j=1}^{n}t_{ij},i=1,2,\ldots ,n$$

(5) $$C_{j}=\sum_{j=1}^{n}t_{ij},i=1,2,\ldots ,n$$

where *D_i_* is the sum of the rows of the corresponding rows in the comprehensive influence matrix *T*; *C_j_* is the sum of the columns of the corresponding columns in the comprehensive impact matrix *T*; and *t_ij_* is the elements of row *i* and column *j* in the comprehensive impact matrix *T*.

(4) Calculate the centrality and cause degree of each influencing factor.

The expression between the centrality of each dimension *M_i_* and the causality degree *R_j_* is

(6) $$M_{i}=D_{i}+C_{j}$$

(7) $$R_{i}=D_{i}-C_{j}$$

### 2.3. Interpretative Structural Modeling

Interpretative structural modeling (ISM) is a system engineering analysis method that can decompose a complex system into several related dimensions. Through matrix operations and graph theory methods, it establishes the hierarchical structure and causal relationships among these dimensions, thereby revealing the inherent logic and hierarchical relationships of the system [14,15]. Based on the results of the DEMATEL method, this paper establishes a hierarchical model of the dimensions of job demand and analyzes the role and dependence of each dimension at different levels, as well as the hierarchy and structure of the whole system. The specific calculation process is as follows:

(1) Calculate the adjacency matrix *A*.

We introduce a threshold *λ* (*λ* ∈ [0, 1]) to simplify the system structure. Based on the comprehensive impact matrix *T*, we calculate the adjacency matrix *A*, where the expression for the elements *a_ij_* in *A* is

(8) $$a_{ij}=\left\{\begin{array}{c} 1,t_{ij}\geq \lambda \\ 0,t_{ij}<\lambda \end{array}\right.$$

(2) Calculate the reachable matrix *M*.

Considering the influence of the dimensions themselves, the adjacency multiplication matrix *B* is calculated as follows:

(9) B=A+I

Multiply the adjacency multiplication matrix *B* to obtain the reachable matrix *M*,

(10) $$M=B^{k+1}=B^{k}\neq B^{k-1}$$

where *k* = 1, 2, …, *n*.

(3) Classify and analyze the hierarchical structure of the dimensions.

We categorize the levels of dimensions and establish a hierarchical structure model, then apply the reachable matrix *M* to each dimension *S* to determine the following set:

(11) $$R\left(S_{i}\right)=\left\{S_{i}|m_{ij}=1\right\}$$

(12) $$A(S_{i})=\left\{S_{j}|m_{ij}=1\right\}$$

(13) $$C\left(S_{i}\right)=R(S_{i})\cap A(S_{i})$$

where *R*(*S_i_*) is the reachable set, the set of elements reachable by *S_i_* in the reachable matrix *M*. *A*(*S_i_*) is the antecedent set, the set of elements that can reach *S_i_* in the reachable matrix *M*; *C*(*S_i_*) is the common set, the intersection of *S_i_* in the reachable set *R*(*S_i_*) and the antecedent set *A*(*S_i_*); and *m_ij_* is the element in the reachable matrix *M*.

We then determine whether the reachable set *R*(*S_i_*) is equal to the common set *C*(*S_i_*), and if it is equal, we can obtain the uppermost unit *B*(*S_i_*) of the system and delete it in the new reachability set. Through the above steps, the system is divided into several levels to obtain the hierarchical structure of dimensions. The higher the level, the more direct the dimensions, and the lower the level, the more the root cause of the dimensions is indicated.

### 2.4. MICMAC Analysis

The method of cross-impact matrix multiplication (MICMAC) was proposed by J.C. Dupuy and M. Godet in 1973. The MICMAC method is a comprehensive hierarchical analysis technology that can be used to analyze the dependency relationship between the dimensions of job demands and show it in the form of coordinate axes [16,17]. The *x*-coordinate represents dependence, while the *y*-coordinate represents driving force.

The core idea of this method is to calculate the driving force value and dependence value between each dimension through the reachable matrix *M* (the specific calculation formula is as follows). Then, the dimensions of job demand are divided into autonomy dimension (III), independence dimension (II), relevance dimension (I), and dependency dimension (IV) [18,19].

(14) $$D_{I}=\sum_{j=1}^{n}t_{ij}(n=1,2,\ldots ,n)$$

(15) $$C_{i}=\sum_{j=1}^{n}t_{ij}(n=1,2,\ldots ,n)$$

## 3. Data Processing and Analysis Results

### 3.1. Sample Selection and Collection

We comprehensively compared factors such as the reliability of reviews information on various employment information websites, the popularity of companies, and the breadth of industries. During the data collection process, we adopted strict selection criteria to ensure the accuracy of data sources and the effectiveness of the analysis. After a meticulous selection process, we ultimately decided to use the Maimai platform as our primary data source. The reasons are as follows. We thoroughly examined multiple potential data sources, including LinkedIn, Zhaopinand, and Maimai platforms. Through comparative analysis, Maimai demonstrated clear advantages in several aspects. As a leading workplace social platform in China, Maimai attracts many new-generation employees, which aligns closely with our research subject. Secondly, Maimai’s user identity verification mechanism and content review process ensure the authenticity and reliability of reviews, which is crucial for our study. Furthermore, the content shared by Maimai users covers various aspects such as work experience, salary and welfare, and career development, all of which are directly related to the dimensions of employee job demands that our research focuses on, providing rich material for in-depth analysis. Finally, the data collected from the Maimai platform can reflect employees’ genuine feelings and evaluations, which is directly relevant for identifying and analyzing employee job demands.

In addition, we selected well-known Internet and technology companies where new-generation employees are concentrated on the Maimai platform as our research subjects, including Tencent (Shenzhen, China), Huawei (Shenzhen, China), New H3C Group (Hangzhou, China), Qihoo 360 (Beijing, China), ByteDance (Beijing, China), Xiaomi (Beijing, China), Microsoft (Redmond, WA, USA), Alibaba (Hangzhou, China), ZTE (Shenzhen, China), Meituan (Beijing, China), JD.com (Beijing, China), Didi (Beijing, China), Baidu (Beijing, China), NetEase (Hangzhou, China), and Hikvision (Hangzhou, China). The rationale for focusing on these companies is multifaceted. Firstly, these companies are the main gathering places for new-generation employees, making them highly representative for studying the work attitudes, career development expectations, and workplace culture of this demographic. Secondly, the new-generation employees in these companies are highly active on professional social platforms, where they generate a significant number of online reviews and discussions, providing a rich resource for our data collection. Furthermore, these companies often operate at the forefront of their industries, and their employees may face work challenges and career development opportunities that are particularly characteristic of our times, which is crucial for exploring the job demands and workplace adaptation issues of new-generation employees. Additionally, these companies have a substantial influence, and studying their new-generation employees can offer deep insights into the professional environment of the Internet and technology sectors in China.

The collected data includes employees’ online reviews of their companies and positions, each ID score, corporate salary and welfare score, personal development score, team atmosphere score, business prospect score, and overall score. The collection period covers review data from November 2021 to 15 June 2024. A total of 1409 pieces of data were collected, as detailed in Table 1.

### 3.2. Data Preprocessing

The data of the employees’ online reviews we collected are not entirely relevant to the research needs, as they contain a large amount of irrelevant information. Therefore, we first performed data cleaning, or “denoising”, by removing duplicate reviews, blank reviews, and so on. Next, we conducted data preprocessing, which mainly includes word segmentation and the removal of stop words. For word segmentation, we employed a Chinese word segmentation method known as “Jieba” and incorporated a custom-built dictionary. The removal of stop words primarily involved eliminating conjunctions, modal particles, transitional words, and so forth. Finally, according to the results, we deleted stop words to provide reliable data support for subsequent text topic mining.

### 3.3. Determine the Number of Topics

In order to determine the optimal number of topics, this paper uses Coherence Score and Perplexity as evaluation indicators to assess the model’s fit and generalization ability under different numbers of topics [20]. The Coherence Score is a measure of the correlation between words within a topic in a topic model, which reflects the semantic similarity of words in a topic, and the Coherence Score reflects the internal quality of a topic [21]. Perplexity is a measure of the predictive ability of a probabilistic model, and a lower Perplexity degree indicates that the model is more predictive of new data and the quality of the model is higher [22,23]. The formula for calculating Perplexity is shown in Equation (16).

(16) $$Perplexity\left(D\right)=exp\left\{-\frac{\sum_{d=1}^{M}logp(w_{d)}}{\sum_{d=1}^{M}N_{d}}\right\}$$

**Figure 3:**
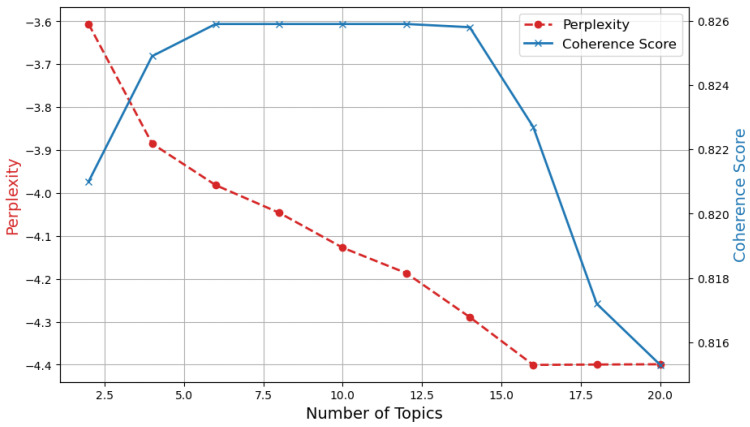
Coherence Score and Perplexity.

The specific results of the analysis of Coherence Score and Perplexity for different subject numbers are shown in Figure 3. As can be seen from Figure 3, Perplexity gradually decreases with the increase in the number of topics but begins to stabilize when the number of topics is 20. The Coherence Score reached its highest value (0.8259) at six subjects and then gradually decreased. Therefore, topic number 10 was chosen as the optimal number of topics because it has one of the highest consistency scores and the confusion is relatively low, indicating a good balance between topic interpretability and model fitting.

### 3.4. Identification of Job Demand Dimensions Based on LDA Analysis

We set the number of topics in the LDA topic model to 10 and obtained the topic and vocabulary” table under 10 topics, as shown in Table 2.

**Figure 4:**
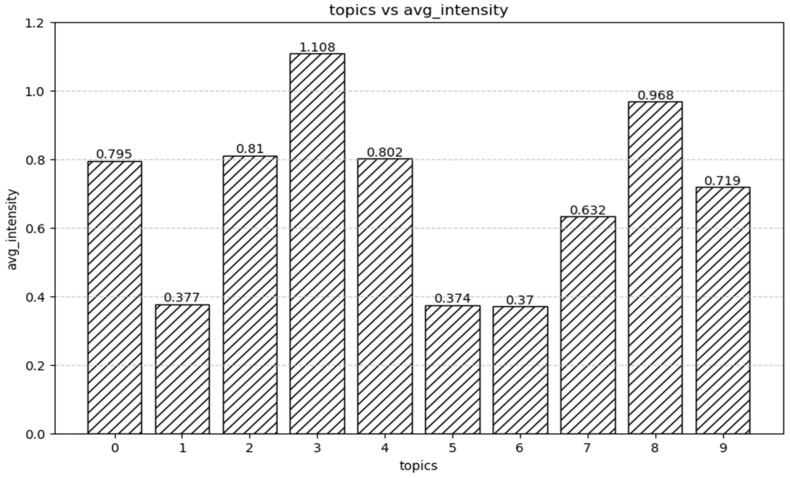
Relative importance of topics.

In addition, based on the distribution of topics in the document, the relative importance of topics is calculated in this paper, and the specific results are shown in Figure 4. The specific calculation process is as follows: ① Obtain the document–topic matrix: each element represents the proportion (or weight) of topic *t* in document *d*, that is, *P*(*t|d*). ② Calculate the average relative strength of each topic: for each topic *t*, calculate its average weight across all documents [24]; the specific formula is

(17) $$RelativeIntensity=\frac{1}{N}\sum_{d=1}^{N}P(\frac{t}{d})$$

where *N* is the total number of documents.

As can be seen from Figure 4, Topic 3 has the highest average relative intensity of 1.108299, which is significantly higher than the other themes. This suggests that Topic 3 dominates in terms of relative strength in this datasets. Topic 8 has the second highest average relative strength at 0.967534. Although it is not as good as Topic 3, it is still significantly higher than other topics. The average relative intensities of Topics 2, 4, and 0 were 0.810466, 0.801789, and 0.795333, which were at a medium level. The relative strength of these three themes is not much different, and the performance is relatively close. The average relative strength of Topics 9 and 7 was 0.718584 and 0.632237, which was significantly lower than the medium but still higher than the weakest theme. The mean relative intensities of Topics 1, 5, and 6 were 0.376712, 0.373682, and 0.370363. The average relative strength of these three themes was the lowest, and their performance was relatively similar and significantly lower than that of the other themes. Therefore, it can be seen that Topic 3 and Topic 8 are significantly higher in average relative intensity than other topics, and, in particular, Topic 3 exceeds Topic 1 in relative intensity. The mean relative intensity ranged from the highest at 1.108299 to the lowest at 0.370363, showing a large difference in intensity distribution between different subjects. From the mean relative intensity, it can be inferred that Topics 3 and 8 may play a more important role in the dataset and deserve further attention and research.

### 3.5. Calculation of Centrality and Cause Degree of Job Demand Dimensions Based on the DEMATEL Model

A total of 38 new-generation practitioners were invited to rate the degree of influence between the dimensions of job demand, including 10 Internet employees, 4 computer/software employees, 4 education/training employees, 3 financial industry employees, and 17 manufacturing employees. The scoring method adopts a 0–4 scoring system, with 0 representing no effect, 1 representing weak effect, 2 representing moderate effect, 3 representing strong effect, and 4 representing very strong effect, resulting in a direct impact matrix, as shown in Equation (18). In order to ensure the reliability of the scoring results, the SPSS27 soft drop was used to test the reliability of the scoring personnel’s opinions, and the value of Cronbach’s *α* was 0.967, which was greater than 0.8, indicating high reliability.

(18) $$A=\left[\begin{array}{cccc} 0 & x_{12} & \cdots & x_{1n}\\ x_{21} & 0 & \ldots & x_{2n}\\ \vdots & \vdots & \ddots & \vdots \\ x_{m1} & x_{m2} & \cdots & 0 \end{array}\right]$$

In order to calculate the centrality and cause degree of each dimension, it is first necessary to normalize the scoring matrix of practitioners according to Equations (1)–(3) and calculate the comprehensive impact matrix *T*, and the calculation results are shown in Table 3. The comprehensive impact matrix is one of the core tools used in the Decision-Making Trial and Evaluation Laboratory (DEMATEL) method. The comprehensive impact matrix is a mathematical matrix that can represent the direct influence intensity between job demand dimensions. This matrix is constructed through the judgments of professionals and is used to quantify the mutual influence among job demand dimensions. By using the comprehensive impact matrix, key dimensions within job demand can be identified, along with the causal relationships between them.

**Figure 5:**
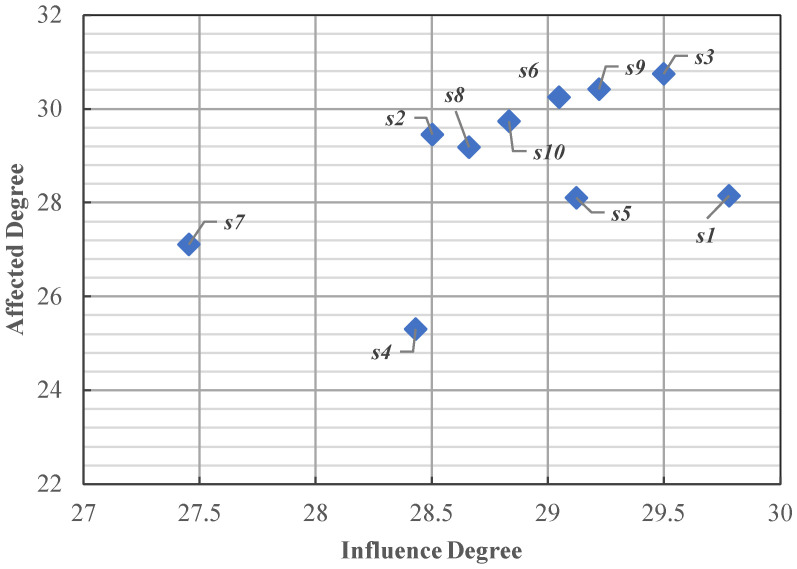
Influence degree and affected degree.

According to Equations (4)–(7), influence degree, affected degree, centrality, and cause degree are calculated, and the calculation results are shown in Table 4 and Figure 5.

In the DEMATEL method, the influence degree usually indicates the degree of influence of a dimension on another dimension. The greater the influence degree, the greater the influence of the dimension on other dimensions. The affected degree indicates how much a dimension is affected by other dimensions, and the greater the affected degree, the more likely it is to be affected or interfered with by other dimensions [25]. As can be seen from Figure 5, the influence degree and affected degree of most of the dimensions are concentrated in a high range, and there are differences in the influence degree and affected degree of different dimensions. Among them, the most influential dimension is leadership and team atmosphere (*s*_1_), with a value of 29.778, indicating that leadership and team atmosphere (*s*_1_) has the greatest impact on the job demand for new-generation employees. It is followed by welfare and promotion (*s*_3_), Corporate prospect and personal development (*s*_9_), overall environment and platform (*s*_5_), and opportunities and resources (*s*_6_). The most affected dimension was welfare and promotion (*s*_3_), with a value of 30.758, indicating that welfare and promotion (*s*_3_) was very susceptible to the influence of other dimensions. However, the influencing values of corporate prospects and personal development (*s*_9_), opportunities and resources (*s*_6_), and work stress and position (*s*_10_) decreased in turn.

**Figure 6:**
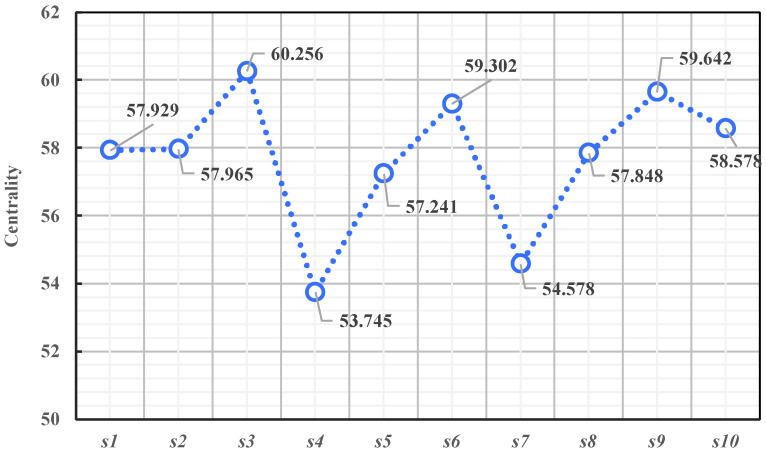
Importance curve of the job demand dimension.

In the DEMATEL method, centrality can reflect the role and influence of one dimension in the overall dimension of job demand, as well as the closeness of its interaction with other dimensions. In the DEMATEL analysis, the higher the centrality of a dimension, the more important it is in the dimension of job demand, and it may have a greater impact on the operation and results of the overall job demand [26]. As can be seen from Table 4 and Figure 6, the dimensions that have the greatest impact on new-generation employees are welfare and promotions (*s*_3_), corporate prospects and personal development (*s*_9_), and opportunities and resources (*s*_6_). This is followed by work stress and position (*s*_10_), working relationship and intensity (*s*_2_), leadership and team atmosphere (*s*_1_), business and industry development (*s*_8_), overall environment and platform (*s*_5_), platforms and technologies (*s*_7_), and company culture and industry (*s*_4_). The results show that welfare and promotion (*s*_3_) and corporate prospect and personal development (*s*_9_) are the core needs of new-generation employees, and they are the dimensions that have the greatest impact on the work of new-generation employees.

**Figure 7:**
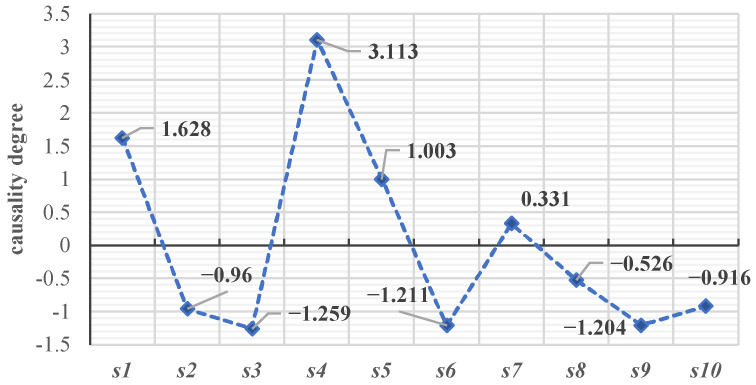
Cause and effect diagram of the job demand dimension.

In the DEMATEL method, the cause degree is used to measure the degree to which a dimension acts as a cause in the dimension of job demand. If the value of the cause degree is greater than zero, it indicates that the dimension affects other dimensions more as a cause. If the value of the cause degree is less than zero, it indicates that the dimension is more affected by other dimensions [27]. According to the cause degree value (as shown in Figure 7), leadership and team atmosphere (*s*_1_), company culture and industry (*s*_4_), overall environment and platform (*s*_5_), and platforms and technologies (*s*_7_) are the cause dimensions of new-generation employees’ job demand that have a greater impact on other dimensions. Working relationships and strength (*s*_2_), welfare and promotions (*s*_3_), opportunities and resources (*s*_6_), business and industry development (*s*_8_), corporate prospects and personal development (*s*_9_), and work stress and position (*s*_10_) are the outcome dimensions of new-generation employees’ work needs that are more susceptible to the influence of other dimensions.

### 3.6. Hierarchical Relationship Division of Job Demand Based on the ISM Model

In the ISM model, the hierarchical division of job demand dimensions is based on the comprehensive impact matrix in Table 3, and the overall impact matrix is calculated according to Equation (8); then, the threshold value *λ* is introduced, and the reachable matrix *F* is obtained by combining with Equation (9), as shown in Table 5. At the same time, the reachable matrix *F* is divided into reachable set, antecedent set, and intersection, and the partition results are shown in Table 5 and Table 6.

The ISM model can show hierarchical relationships and dependencies between dimensions of job demand [28,29]. As can be seen from Table 7, in the dimensions of the job demand of new-generation employees, working relationship and intensity (*s*_2_), welfare and promotion (*s*_3_), opportunities and resources (*s*_6_), business and industry development (*s*_8_), corporate prospects and personal development (*s*_9_), and work stress and position (*s*_10_) are at the top level, and there is a relationship between the two to influence and restrict each other. Company culture and industry (*s*_4_), overall environment and platform (*s*_5_), and platforms and technologies (*s*_7_) are in the middle layer, which has a direct impact on the first layer and is the shallow dimension of the work needs of new-generation employees. Leadership and team atmosphere (*s*_1_) is at the bottom, which is the deepest dimension of the job demands of new-generation employees, has a direct or indirect impact on other dimensions through different ways, and is the most fundamental demand of the work needs of new-generation employees.

### 3.7. Dimensional Driving Force and Dependency Analysis of Work Requirements Based on the MICMAC Model

In MICMAC analysis, driving power and dependence can explain the role and interrelationship of each dimension in the dimensions of job demand. Driving power refers to the degree to which one dimension influences other dimensions in job demand dimensions. The dimensions with high driving power are usually located in the upper layer of job demand dimensions, and they are the main driving power in job demand dimensions and can have a greater impact on other dimensions. Dependence refers to the degree to which a dimension is affected by other dimensions in job demand dimensions. dimensions with high dependence are usually lower than job demand dimensions, and they are more affected by the other dimensions than the others [30,31]. According to Equations (14) and (15), the driving power and dependence of job demand dimensions can be calculated, as shown in Table 8. As can be seen from Table 8, leadership and team atmosphere (*s*_1_), company culture and industry (*s*_4_), and overall environment and platform (*s*_5_) have higher drivers, indicating that they are the main driving powers, while they are less dependent, indicating that they are less influenced by other dimensions. While working relationships and intensity (*s*_2_), opportunities and resources (*s*_6_), business and industry development (*s*_8_), corporate prospects and personal development (*s*_9_), and work stress and positions (*s*_10_) have lower driving powers and higher dependence, suggesting that they are more influenced by other dimensions, welfare and promotion (*s*_3_) have both higher driving force and higher dependency, indicating that this dimension is both a driving force and a dependence dimension. The dimension of platforms and technologies (*s*_7_) has a lower driving power and dependence, indicating that its role is relatively independent.

**Figure 8:**
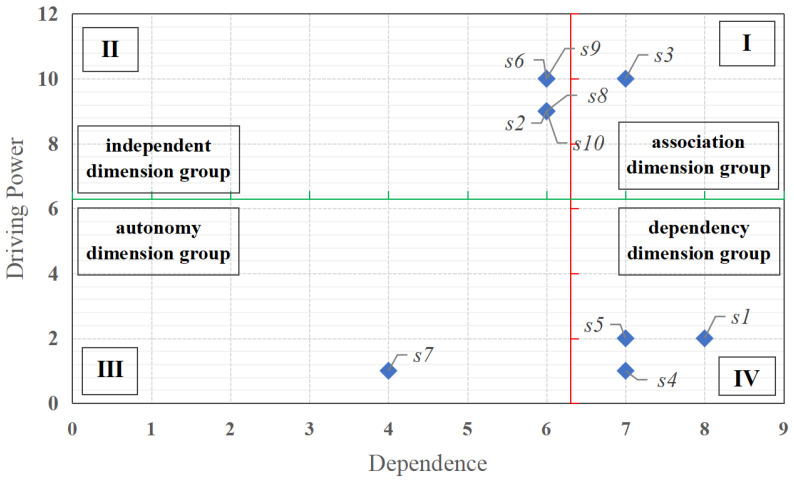
Diagram of driving power and dependence in the dimensions of job demand.

According to the driving power and dependence values calculated in Table 8, the diagram of driving power and dependence for job demand dimensions was plotted with the dependence value as the abscissa and the driving power value as the ordinate (Figure 8). According to Figure 8, there are nine dimensions with high dependence, indicating that most of the job demand dimensions are easily affected by other dimensions, the dependence between dimensions is relatively high, and the role between different dimensions is more obvious, so we should pay attention to the correlation between dimensions. Among them, the autonomy dimension group (III) contains one dimension, which is only platforms and technologies (*s*_7_), indicating that platforms and technologies (*s*_7_) have little impact on the overall job demand. The dependency dimension group (IV) contains three dimensions, namely leadership and team atmosphere (*s*_1_), company culture and industry (*s*_4_), and overall environment and platform (*s*_5_), indicating that these dimensions are more dependent on other dimensions and are more obviously affected by other dimensions. At the same time, these have certain limitations on their role in other dimensions, so the dimensions located in the dependent dimension group (IV) need more attention. The association dimension group (I) contains one dimension, which is only welfare and promotion (*s*_3_), which has a high driving power and dependence and is also susceptible to other dimensions. The independent dimension group (II) contains four dimensions, namely working relationship and intensity (*s*_2_), opportunity and resources (*s*_6_), business and industry development (*s*_8_), corporate prospect and personal development (*s*_9_), and work stress and position (*s*_10_); these dimensions have strong driving power and weak dependence, which are the key dimensions that affect the internal collaboration of job demand dimensions and can have a positive effect on the job enthusiasm of new-generation employees through the management of these dimensions.

## 4. Discussion

With the influx of new-generation employees, organizations are faced with increasingly complex and diverse job demands. In order to comprehensively understand and meet the needs of these employees, we employed an integrated model of LDA-DEMATEL-ISM-MICMAC and conducted an in-depth analysis of various dimensions of job demands based on online comments from employees.

Based on LDA analysis, we extracted 10 themes from the online comments of new-generation employees, covering dimensions such as leadership and team atmosphere (*s*_1_), working relationship and intensity (*s*_2_), welfare and promotion (*s*_3_), company culture and industry (*s*_4_), overall environment and platform (*s*_5_), opportunities and resources (*s*_6_), platforms and technologies (*s*_7_), business and industry development (*s*_8_), corporate prospects and personal development (*s*_9_), and work stress and position (*s*_10_). Through the analysis of influence degree, affected degree, centrality, and cause degree, we gained in-depth understanding of the interrelationships and influences among these dimensions. Additionally, we utilized the ISM model and the MICMAC model to analyze the hierarchical structure and influence of these dimensions, further revealing the key driving factors and interdependencies of job demands for the new-generation employees.

Based on our research findings, several key conclusions can be drawn. Firstly, leadership and team atmosphere (*s*_1_) has been identified as the most influential dimension in the job demands of new-generation employees. Our study reveals that the behavior of leaders and the team culture directly impact employee work attitudes and behaviors. Therefore, organizations should focus on developing effective leadership practices and fostering a positive team atmosphere to meet the job demands of this generation.

Secondly, welfare and promotion (*s*_3_) has been identified as the most affected dimension. This indicates that the new generation employees places significant importance on aspects such as welfare, promotion, and career prospects. Companies should ensure the effectiveness and fairness of their welfare and promotion policies to maintain employee satisfaction and loyalty.

Additionally, our analysis highlights the core needs of new-generation employees, which include welfare and promotion (*s*_3_), as well as corporate prospects and personal development (*s*_9_). These dimensions have a decisive impact on overall job demands and play a key role in meeting the unique needs of this generation. Organizations should prioritize these dimensions in their human resource strategies to attract, motivate, and retain new-generation employees.

Moreover, our findings demonstrate that leadership and team atmosphere (*s*_1_), company culture and industry (*s*_4_), overall environment and platform (*s*_5_), and platforms and technologies (*s*_7_) have a significant role in promoting the job demand of new-generation employees. These factors are critical drivers influencing employee job satisfaction and performance. On the other hand, dimensions such as working relationship and intensity (*s*_2_), welfare and promotions (*s*_3_), opportunities and resources (*s*_6_), business and industry development (*s*_8_), corporate prospects and personal development (*s*_9_), and work stress and position (*s*_10_) are more reflective of the impact on the job demand of new-generation employees, which are the result of being driven by other dimensions.

In conclusion, we emphasize the importance of leadership, team atmosphere, welfare, promotion, corporate prospects, and personal development in meeting the job demands of new-generation employees. By understanding and effectively addressing these dimensions, organizations can create a work environment that aligns with the expectations and aspirations of the new generation, leading to increased satisfaction, productivity, and organizational success.

## 5. Conclusions and Management Implications

### 5.1. Conclusions

We employed the integrated model of LDA-DEMATEL-ISM-MICMAC to analyze the dimensions of job demand for new-generation employees. The significance of our study lies not only in helping organizations better understand and address the needs of new-generation employees but also in contributing to the refinement and expansion of existing motivation theories, providing more effective theoretical guidance for the practice of human resource management. By conducting in-depth research on the dimensions of job demands for new-generation employees, we can provide tailored human resource strategies for organizations to enhance employee satisfaction, work efficiency, and organizational performance. This is not only an important competitive advantage for organizations but also a key factor in achieving sustainable development.

The contributions of our study are as follows. ① Traditional studies on dimensions of job needs are primarily based on Maslow’s Hierarchy of Needs theory and employing theoretical deductions and empirical research for analysis. We adopt a complex combined model (LDA-DEMATEL-ISM-MICMAC), integrating text mining, causal relationship analysis, structural modeling, and matrix analysis, providing a data-driven research approach. ② Maslow’s theory divides needs into five levels, while this paper extracts ten specific topics of job demands from online reviews using the LDA model and offers a more detailed understanding of needs dimensions. ③ Maslow’s Hierarchy of Needs theory emphasizes the progression of needs from lower to higher levels. By contrast, we analyzed the dynamic interactions between different needs dimensions using the DEMATEL and ISM models and revealed the causal relationships and hierarchical structure among these dimensions. ④ Through the DEMATEL model, we identified that leadership and team atmosphere are key factors influencing the work needs of new-generation employees, whereas Maslow’s theory does not explicitly distinguish specific influencing factors. ⑤ We further classified and analyzed needs dimensions using the MICMAC model, revealing the driving forces and dependencies among different needs dimensions, in contrast to Maslow’s theory, which does not provide such a detailed multi-dimensional analysis framework. In summary, the combined model of LDA-DEMATEL-ISM-MICMAC based on employees’ online reviews in this paper offers a new perspective and method for analyzing the work needs of new-generation employees, addressing the shortcomings of Maslow’s traditional Hierarchy of Needs theory in refining needs dimensions and analyzing the dynamic interactions of needs.

### 5.2. Management Implications

To effectively address the job demands of new-generation employees, several management insights are proposed based on the above conclusions.

(1) Strengthening leadership and team atmosphere. In view of the importance of leadership and team atmosphere (*s*_1_) in the work needs of new-generation employees, companies should attach great importance to the cultivation of leadership and the construction of team culture. First, a company should establish a systematic leadership training system, covering communication skills, conflict management, emotional intelligence, etc., to improve the management’s communication skills, motivation, and team management capabilities. Secondly, the company should organize regular team-building activities to enhance interactions and trust between team members. In addition, the company should create an open, inclusive, and positive work atmosphere, encouraging employees to express their opinions freely so that employees’ ideas and feelings are valued. At the same time, the company should work with team members to develop clear work goals and visions, ensuring that each member understands their roles and contributions, and establish a reasonable incentive mechanism to reward and recognize team and individual performance.

(2) Optimizing welfare and promotion mechanisms. Policies for welfare and promotions (*s*_3_) not only directly affect the job satisfaction of employees but are also susceptible to other factors, so companies should take comprehensive measures to ensure their effectiveness and attractiveness. Companies need to regularly review and adjust their welfare policies to ensure that they are fair and competitive, make timely adjustments based on employee feedback, and ensure that they are in line with the needs of employees and changes in the work environment. At the same time, companies should establish a transparent promotion mechanism and clarify the career development path of employees to help them see future growth opportunities and enhance employees’ sense of belonging and loyalty. Companies should hold regular career development seminars and training courses to help employees improve the skills and qualities they need, while providing career coaching and guidance to help them develop personal development plans. In addition, to strengthen employees’ trust in the promotion mechanism, leaders are encouraged to have regular career development conversations with team members to keep abreast of their career expectations and development needs and to ensure that their employees feel supported and cared for in their careers.

(3) Focusing on corporate prospects and personal development. The trajectories of company prospects and personal development play a vital role in shaping the future of next-generation employees. These paths directly impact their long-term career planning and personal growth. Firstly, it is crucial for a company to effectively communicate its development vision and strategic goals. This can ensure that employees gain a comprehensive understanding of the company’s future direction and market positioning. By conducting all-hands meetings and implementing internal communication strategies that articulate the company’s vision, mission, and core values, the company can ensure that every employee feels valued and significant in the company’s growth journey.

Secondly, companies should provide a wealth of career development opportunities, including training, project experience, and career guidance, to help employees improve their skills and professionalism. At the same time, employees are encouraged to participate in industry exchanges and external training to broaden their horizons and enhance their professional capabilities. Companies should establish an internal knowledge-sharing platform to encourage employees to share learning resources and experiences. To ensure that employees have unobstructed access to the tools and information they need, companies should regularly evaluate workflows and resource allocation and adjust and optimize them in a timely manner. This not only improves work efficiency but also makes employees feel valued and cared for by the company. Finally, companies should have regular career development conversations with employees to understand their expectations and needs and give timely feedback and suggestions. This open communication helps to build a good relationship of trust and makes employees feel that the company cares and supports their career development.

(4) Improving the working environment and providing resources. The overall environment and platforms (*s*_5_) and opportunities and resources (*s*_6_) dimensions indicate that they play an important role in meeting the needs of employees. By investing in improving the working environment and allocating resources, companies can regularly assess the comfort and safety of the work environment. It is crucial to ensure that the office space adheres to the principles of humanized design, which includes optimizing the office layout, enhancing lighting and ventilation, and providing comfortable office furniture. Creating a pleasant working atmosphere will not only improve work efficiency and productivity but also enhance employee satisfaction.

(5) Management of work pressure. The high impact of work stress and positions (*s*_10_) suggests that work stress can be influenced by various factors. Therefore, it is essential for companies to pay attention to the workload of employees, ensuring that work tasks are reasonably assigned and that each employee’s workload remains within a manageable range. This proactive approach helps to prevent stress and fatigue caused by an excessive workload. Simultaneously, companies can optimize employee well-being by offering stress management training, mental health support, and flexible work arrangements. These initiatives aim to equip employees with stress management skills and coping strategies, including time management, emotion regulation, and relaxation techniques. By addressing employees’ safety and social and esteem needs, companies can ensure that their workforce remains in good physical and mental condition.

## 6. Limitations and Future Directions

We have achieved valuable results by analyzing the dimensions of job demands for new-generation employees based on online employee comments. However, our research also has some limitations. Firstly, our study heavily relies on data from the Maimai platform, which may introduce biases, as the user demographics and content may differ from other platforms, limiting the generalizability of the research findings and excluding employees with different demographic characteristics or industries. Secondly, our research sample was primarily focused on the information technology and Internet industry, which might have led to the overemphasis of industry-specific characteristics, while the traits of other industries might not have been fully represented. Thirdly, the time frame of data collection spanned from November 2021 to June 2024, providing insights into recent trends, but this might not have captured long-term changes or emerging factors that influence job demands. Additionally, our study focused on new-generation employees in China, and the findings may not directly apply to employees from other cultural backgrounds, as different values and expectations in other cultural contexts may influence job demands.

Future research should aim to integrate data from various platforms and across different industries in order to achieve a more holistic understanding of job requirements for the next generation of workers. Furthermore, broadening the sample to include a diverse range of industries, company sizes, and geographical areas will significantly enhance the generalizability and applicability of this study’s findings. Additionally, longitudinal studies can be conducted to track the changes in job demands over time and identify emerging trends. Exploring the job demands of employees from different cultural backgrounds can provide a deeper understanding of the factors influencing new-generation employees globally.

## Figures and Tables

**Table 1:** Basic information on the review data.

Company Name	Number of Comments	Salary and Welfare Scores	Personal Development Score	Team Atmosphere Score	Business Prospect Score	Overall Score
Tencent	234	4.2	4.1	4.2	4.1	4.2
Huawei	164	4.3	4.2	4.2	4.2	4.2
New H3C Group	76	3.9	3.9	4.0	3.9	3.9
Qianxin	137	3.7	3.7	3.8	3.7	3.7
ByteDance	145	4.3	4.2	4.2	4.2	4.2
Xiaomi	98	3.9	3.9	4.0	3.9	4.0
Microsoft Corporation	39	4.2	4.1	4.2	4.1	4.2
Alibaba	91	4.3	4.2	4.2	4.1	4.2
ZTE	115	4.0	3.9	4.1	4.0	4.0
Meituan	88	4.0	4.0	4.0	4.0	4.0
JD	51	4.1	4.0	4.1	4.1	4.1
Didi	55	4.2	4.1	4.1	4.1	4.1
Baidu	54	4.1	4.0	4.0	4.0	4.0
NetEase	23	4.0	4.0	4.1	4.0	4.1
Hikvision	30	3.9	3.9	4.0	4.0	4.0
average value		4.1	4.0	4.1	4.0	4.1

**Table 2:** Topic–vocabulary.

Numbering	Topic	Vocabulary
0	Leadership and Team atmosphere (*s*_1_).	Leadership, team atmosphere, salary, welfare, department, space, overall, promotion, large factory, employees
1	Working Relationship and Intensity (*s*_2_).	Colleagues, team atmosphere, leadership, technology, process, office, relationship, garbage, boss, work intensity
2	Welfare and Promotions (*s*_3_).	Welfare, salary, provident fund, year-end bonus, business, big factory, team atmosphere, layoffs, promotion, peak
3	Company Culture and Industry (*s*_4_).	Company, team atmosphere, welfare, projects, employees, capabilities, salary, salary, Internet, industry
4	Overall Environment and Platform (*s*_5_).	Overall, company, atmosphere, team atmosphere, department, platform, business, boss, system, thing
5	Opportunities and Resources (*s*_6_).	Opportunity, working atmosphere, experience, treatment, platform, resources, welfare, spicy chicken, business, department
6	Platforms and Technologies (*s*_7_).	Platform, culture, business, screws, supervisors, values, technology, process, mechanism, market
7	Business and Industry development (*s*_8_).	Team atmosphere, welfare, salary, personal growth, enterprise, Internet, PUA, industry, business development, technology
8	Corporate Prospects and Personal development (*s*_9_).	Team atmosphere, business, prospects, salary, company, welfare, personal development, department, feeling, overall
9	Work Stress and Position (*s*_10_).	Company, employees, team atmosphere, welfare, pressure, salary, department, Internet, good company, position

**Table 3:** Comprehensive impact matrix.

Factor	*s*_1_	*s*_2_	*s*_3_	*s*_4_	*s*_5_	*s*_6_	*s*_7_	*s*_8_	*s*_9_	*s*_10_
*s*_1_	2.817	3.055	3.186	2.626	2.913	3.129	2.806	3.022	3.154	3.07
*s*_2_	2.79	2.823	3.052	2.508	2.779	3	2.685	2.89	3.016	2.958
*s*_3_	2.887	3.028	3.05	2.594	2.883	3.107	2.781	2.991	3.128	3.05
*s*_4_	2.789	2.907	3.036	2.419	2.78	2.985	2.68	2.887	3.003	2.942
*s*_5_	2.855	2.977	3.118	2.563	2.752	3.06	2.751	2.957	3.078	3.01
*s*_6_	2.843	2.973	3.111	2.562	2.845	2.954	2.741	2.944	3.072	3.003
*s*_7_	2.683	2.817	2.932	2.412	2.688	2.891	2.503	2.79	2.899	2.839
*s*_8_	2.793	2.932	3.06	2.523	2.81	3.024	2.706	2.811	3.032	2.969
*s*_9_	2.861	2.995	3.128	2.571	2.852	3.075	2.76	2.966	2.988	3.022
*s*_10_	2.831	2.955	3.085	2.539	2.817	3.031	2.712	2.928	3.052	2.882

**Table 4:** Calculation results of each dimension.

Factor	Influence Degree	Affected Degree	Centrality	Cause Degree	Dimension Attributes
*s*_1_	29.778	28.15	57.929	1.628	Cause dimension
*s*_2_	28.502	29.463	57.965	−0.96	Outcome dimensions
*s*_3_	29.499	30.758	60.256	−1.259	Outcome dimensions
*s*_4_	28.429	25.316	53.745	3.113	Cause dimension
*s*_5_	29.122	28.119	57.241	1.003	Cause dimension
*s*_6_	29.046	30.256	59.302	−1.211	Outcome dimensions
*s*_7_	27.454	27.123	54.578	0.331	Cause dimension
*s*_8_	28.661	29.187	57.848	−0.526	Outcome dimensions
*s*_9_	29.219	30.423	59.642	−1.204	Outcome dimensions
*s*_10_	28.831	29.747	58.578	−0.916	Outcome dimensions

**Table 5:** Reachable matrices.

Factor	*s*_1_	*s*_2_	*s*_3_	*s*_4_	*s*_5_	*s*_6_	*s*_7_	*s*_8_	*s*_9_	*s*_10_
*s*_1_	1	1	1	0	1	1	0	1	1	1
*s*_2_	0	1	1	0	0	1	0	1	1	1
*s*_3_	1	1	1	0	0	1	0	1	1	1
*s*_4_	0	1	1	1	0	1	0	1	1	1
*s*_5_	0	1	1	0	1	1	0	1	1	1
*s*_6_	0	1	1	0	0	1	0	1	1	1
*s*_7_	0	0	1	0	0	1	1	0	1	0
*s*_8_	0	1	1	0	0	1	0	1	1	1
*s*_9_	0	1	1	0	0	1	0	1	1	1
*s*_10_	0	1	1	0	0	1	0	1	1	1

**Table 6:** Dimension set of the reachability matrix.

Factor	Reachable Set *R*	Antecedent Set *Q*	Intersection *A* = *R* ∩ *Q*
*s*_1_	*s*_1_, *s*_2_, *s*_3_, *s*_5_, *s*_6_, *s*_8_, *s*_9_, *s*_10_	*s*_1_, *s*_3_	*s*_1_, *s*_3_
*s*_2_	*s*_2_, *s*_3_, *s*_6_, *s*_8_, *s*_9_, *s*_10_	*s*_1_, *s*_2_, *s*_3_, *s*_4_, *s*_5_, *s*_6_, *s*_8_, *s*_9_, *s*_10_	*s*_2_, *s*_3_, *s*_6_, *s*_8_, *s*_9_, *s*_10_
*s*_3_	*s*_1_, *s*_2_, *s*_3_, *s*_6_, *s*_8_, *s*_9_, *s*_10_	*s*_1_, *s*_2_, *s*_3_, *s*_4_, *s*_5_, *s*_6_, *s*_7_, *s*_8_, *s*_9_, *s*_10_	*s*_1_, *s*_2_, *s*_3_, *s*_6_, *s*_8_, *s*_9_, *s*_10_
*s*_4_	*s*_2_, *s*_3_, *s*_4_, *s*_6_, *s*_8_, *s*_9_, *s*_10_	*s*_4_	*s*_4_
*s*_5_	*s*_2_, *s*_3_, *s*_5_, *s*_6_, *s*_8_, *s*_9_, *s*_10_	*s*_1_, *s*_5_	*s*_5_
*s*_6_	*s*_2_, *s*_3_, *s*_6_, *s*_8_, *s*_9_, *s*_10_	*s*_1_, *s*_2_, *s*_3_, *s*_4_, *s*_5_, *s*_6_, *s*_7_, *s*_8_, *s*_9_, *s*_10_	*s*_2_, *s*_3_, *s*_6_, *s*_8_, *s*_9_, *s*_10_
*s*_7_	*s*_3_, *s*_6_, *s*_7_, *s*_9_	*s*_7_	*s*_7_
*s*_8_	*s*_2_, *s*_3_, *s*_6_, *s*_8_, *s*_9_, *s*_10_	*s*_1_, *s*_2_, *s*_3_, *s*_4_, *s*_5_, *s*_6_, *s*_8_, *s*_9_, *s*_10_	*s*_2_, *s*_3_, *s*_6_, *s*_8_, *s*_9_, *s*_10_
*s*_9_	*s*_2_, *s*_3_, *s*_6_, *s*_8_, *s*_9_, *s*_10_	*s*_1_, *s*_2_, *s*_3_, *s*_4_, *s*_5_, *s*_6_, *s*_7_, *s*_8_, *s*_9_, *s*_10_	*s*_2_, *s*_3_, *s*_6_, *s*_8_, *s*_9_, *s*_10_
*s*_10_	*s*_2_, *s*_3_, *s*_6_, *s*_8_, *s*_9_, *s*_10_	*s*_1_, *s*_2_, *s*_3_, *s*_4_, *s*_5_, *s*_6_, *s*_8_, *s*_9_, *s*_10_	*s*_2_, *s*_3_, *s*_6_, *s*_8_, *s*_9_, *s*_10_

**Table 7:** Hierarchical decomposition table.

Level	Element
Level 1 (Top Floor)	*s*_2_, *s*_3_, *s*_6_, *s*_8_, *s*_9_, *s*_10_
Tier 2	*s*_4_, *s*_5_, *s*_7_
Level 3 (Ground Floor)	*s*_1_

**Table 8:** Driving powers and dependence values.

Factor	Driving Power	Dependence	Factor	Driving Power	Dependence
*s*_1_	8	2	*s*_6_	6	10
*s*_2_	6	9	*s*_7_	4	1
*s*_3_	7	10	*s*_8_	6	9
*s*_4_	7	1	*s*_9_	6	10
*s*_5_	7	2	*s*_10_	6	9

## Data Availability

Data will be made available by the authors upon reasonable request.
